# PREvalence Study on Surgical COnditions (PRESSCO) 2020: A Population-Based Cross-Sectional Countrywide Survey on Surgical Conditions in Post-Ebola Outbreak Sierra Leone

**DOI:** 10.1007/s00268-022-06695-7

**Published:** 2022-09-06

**Authors:** Jurre van Kesteren, Alex J. van Duinen, Foday Marah, Diede van Delft, Antoinette L. Spector, Laura D. Cassidy, Reinou S. Groen, Sonnia-Magba Bu-Buakei Jabbi, Silleh Bah, James A. Medo, Abubakarr Kamanda-Bongay, Daniel van Leerdam, Josien Westendorp, Hanna M. Mathéron, Giulia L. E. Mönnink, Jonathan Vas Nunes, Karel C. Lindenbergh, Sara K. Hoel, Sofie M. Løvdal, Mia N. Østensen, Helene Solberg, Daniel Boateng, Kerstin Klipstein-Grobusch, Daan van Herwaarden, Janine P. J. Martens, H. Jaap Bonjer, Osman Sankoh, Martin P. Grobusch, Håkon A. Bolkan, for the PRESSCO 2020 study group, Jurre van Kesteren, Alex J. van Duinen, Diede van Delft, Laura D. Cassidy, Reinou S. Groen, Sonnia-Magba Bu-Buakei Jabbi, Silleh Bah, James A. Medo, Abubakarr Kamanda-Bongay, Daniel van Leerdam, Josien Westendorp, Hanna M. Mathéron, Giulia L. E. Mönnink, Jonathan Vas Nunes, Karel C. Lindenbergh, Sara K. Hoel, Sofie M. Løvdal, Mia N. Østensen, Helene Solberg, Daniel Boateng, Kerstin Klipstein-Grobusch, Daan van Herwaarden, Janine P. J. Martens, Osman Sankoh, Martin P. Grobusch, Håkon A. Bolkan

**Affiliations:** 1 Amsterdam UMC Location Vrije Universiteit, Department of Surgery, De Boelelaan 1117, 1081 HV Amsterdam, The Netherlands; 2 Global Surgery Amsterdam, Amsterdam, The Netherlands; 3 Institute of Clinical and Molecular Medicine, Norwegian University of Science and Technology (NTNU), Trondheim, Norway; 4 Clinic of Surgery, St. Olavs Hospital HF, Trondheim University Hospital, Trondheim, Norway; 5 CapaCare, Trondheim, Norway; 6 Masanga Hospital, Tonkolili District, Masanga, Sierra Leone; 7 Masanga Medical Research Unit, Tonkolili District, Masanga, Sierra Leone; 8 Institute for Health & Equity and Epidemiology Division, Medical College of Wisconsin, Milwaukee, WI USA; 9 Johns Hopkins School of Medicine, Baltimore, USA; 10 SOS – Surgeons OverSeas, New York, NY USA; 11 Statistics Sierra Leone, Tower Hill, Freetown, Sierra Leone; 12 KIT, Royal Tropical Institute, Amsterdam, The Netherlands; 13 Faculty of Medicine, Vrije Universiteit Amsterdam, Amsterdam, The Netherlands; 14 Julius Global Health, Julius Centre for Health Sciences and Primary Care, University Medical Centre Utrecht, Utrecht University, Utrecht, The Netherlands; 15 University Medical Centre Utrecht, Utrecht, The Netherlands; 16 Amsterdam UMC location University of Amsterdam, AMC, Centre of Tropical Medicine and Travel Medicine, Amsterdam, The Netherlands; 17 School of Public Health, Faculty of Health Sciences, University of the Witwatersrand, Johannesburg, South Africa; 18 Heidelberg Institute of Global Health, University of Heidelberg Medical School, Heidelberg, Germany; 19 Institute of Tropical Medicine, University of Tübingen, Tübingen, Germany; 20 Centre de Recherches Médicales en Lambaréné (CERMEL), Lambaréné, Gabon; 21 Institute of Infectious Diseases and Molecular Medicine (IDM), University of Cape Town, Cape Town, South Africa

## Abstract

**Background:**

Understanding the burden of diseases requiring surgical care at national levels is essential to advance universal health coverage. The PREvalence Study on Surgical COnditions (PRESSCO) 2020 is a cross-sectional household survey to estimate the prevalence of physical conditions needing surgical consultation, to investigate healthcare-seeking behavior, and to assess changes from before the West African Ebola epidemic.

**Methods:**

This study (ISRCTN: 12353489) was built upon the Surgeons Overseas Surgical Needs Assessment (SOSAS) tool, including expansions. Seventy-five enumeration areas from 9671 nationwide clusters were sampled proportional to population size. In each cluster, 25 households were randomly assigned and visited. Need for surgical consultations was based on verbal responses and physical examination of selected household members.

**Results:**

A total of 3,618 individuals from 1,854 households were surveyed. Compared to 2012, the prevalence of individuals reporting one or more relevant physical conditions was reduced from 25 to 6.2% (95% CI 5.4–7.0%) of the population. One-in-five conditions rendered respondents unemployed, disabled, or stigmatized. Adult males were predominantly prone to untreated surgical conditions (9.7 *vs.* 5.9% women; *p* < 0.001). Financial constraints were the predominant reason for not seeking care. Among those seeking professional health care, 86.7% underwent surgery.

**Conclusion:**

PRESSCO 2020 is the first surgical needs household survey which compares against earlier study data. Despite the 2013–2016 Ebola outbreak, which profoundly disrupted the national healthcare system, a substantial reduction in reported surgical conditions was observed. Compared to one-time measurements, repeated household surveys yield finer granular data on the characteristics and situations of populations in need of surgical treatment.

**Supplementary Information:**

The online version contains supplementary material available at 10.1007/s00268-022-06695-7.

## Introduction

Understanding the surgical disease burden and care options available for patients in low- and middle-income countries (LMICs) is vital to raise awareness on the magnitude of treatable conditions and to allocate resources for safeguarding essential surgical services [1].

According to the 2012 Sierra Leone Surgeons Overseas Surgical Needs Assessment (SOSAS), 25% of respondents indicated that they were in need of surgical care at the time of the interview [2]. Since 2012, Sierra Leone has experienced shocks to its health system, most notably the 2013–2016 West African Ebola Virus Disease (EVD) outbreak during which healthcare provision was disrupted, and health-seeking behavior severely altered [3–6]. However, there have also been several major interventions strengthening the health system in Sierra Leone during the past decade. Examples within the surgical domain include the expansion of a surgical task-sharing training program [7, 8], employment of regionally hired medical doctors to support first-level hospitals [9, 10], implementation of a health finance protection scheme [11], and the establishment of national ambulance and emergency services [12].

The PREvalence Study on Surgical COnditions 2020 (PRESSCO 2020) is a replication of the the SOSAS survey [2] and expanded with additional survey topics and a physical examination. The aim of PRESSCO 2020 was to assess the prevalence of physical conditions needing surgical attention and changes over the past decade in Sierra Leone. The type of surgical condition and associated anatomical location, health-seeking behavior, and reasons for not receiving surgical care were also investigated.

## Methods

Located in West Africa, Sierra Leone has a population of 8.3 million and is considered a low-income country [13, 14]. Between October 2019 and March 2020, we conducted a population-based cross-sectional household survey in Sierra Leone.

### Sample size calculation

Sample size calculation was identical with the SOSAS study [2], resulting in an estimated sample size of 3745 individuals. Out of a total of 9671 nationwide clusters, the lowest administrative unit, Statistics Sierra Leone (Stats SL), the governmental institution in charge of statistics and census data, sampled 75 enumeration areas (EA). To select an EA with a probability proportional to the population size, a weighted random cluster design was used. All EAs were located using a global positioning system. Within each cluster, all the households were counted, and 25 households were randomly selected. For each household, a random calculator assigned two household members. A household was defined as one or more people, living together, sharing meals and sleeping in the same structure the night before the interview. If a selected household member was not present, the household was visited up to four times before assigning another household member.

### Study design

PRESSCO 2020 applied the validated SOSAS tool [2, 15]. Based on the self-critique that all data were previously obtained after a verbal examination only, a physical examination was added. This examination was performed if a groin hernia was suspected, when a wound had a diameter of ≥ 5 cm, or was present ≥ 1 month, and to assess the fundal height of pregnant women. Other local adaptions included additional questions on five disease-specific survey topics and blood pressure measurements [16]. Findings from the additional disease specific health topics will be reported in detail separately.

The first section of the survey included demographic information about all household members, surgical procedures, and deaths for any household member during the past year. For the second section, two randomly selected household members were interviewed on surgical conditions covering: face, head, and neck; chest and breast; abdomen; groin, genitals, and buttocks; back; and extremities. The ‘need for surgical care’ labeling depended on the respondent’s judgment. Need for major surgery referred to conditions that required regional or general anesthesia; other procedures were categorized minor surgery. A referral and transport were arranged when medical conditions in need of immediate attention were diagnosed.

Data were collected by nurses, surgically trained community health officers (SACHO) [8] and employees of Stats SL with previous experience from collecting data for the Sierra Leone Demographic Health Survey. All had Sierra Leonean nationality and collectively received a four-day training, followed by an individual assessment prior to the start of the data collection. Physical examination was performed by SACHOs only. Password protected tablets with mobile internet and software application REDCap (Research Electronic Data Capture; Vanderbilt University, Nashville, TN, USA) were used [17, 18]. At two occasions, retrieved data were checked for completeness and potential flaws; prior to departure from the EA by a field supervisor, and after it was uploaded to a cloud-based server by the study group. Traditional leaders provided community approval. Informed consent was obtained from all respondents or parents in cases of children.

### Statistical analysis

Household and individual respondent characteristics were described using central tendency measures, frequencies, and percentages. Findings were compared against earlier study data from 2012 [2]. Bivariate associations were analyzed with Chi-square tests for contingency tables and* t*-tests for normally distributed data. All tests were conducted as two-tailed, and statistical significance was set at *p* < 0.05. Stata® 15.1 (StataCorp, College Station, Texas, USA) and Microsoft Excel® (Microsoft, Redmond, Washington, USA) were used.

### Ethics

The study was registered with the ISRCTN registry (No. 12353489). Ethics approval was obtained from the Norwegian Regional Committee for Medical and Health Research Ethics (REC (2019/31932), and Sierra Leone Ethics and Scientific Review Committee (SLESRC 2019/October/03).

## Results

### Enumeration areas

**Fig. 1:**
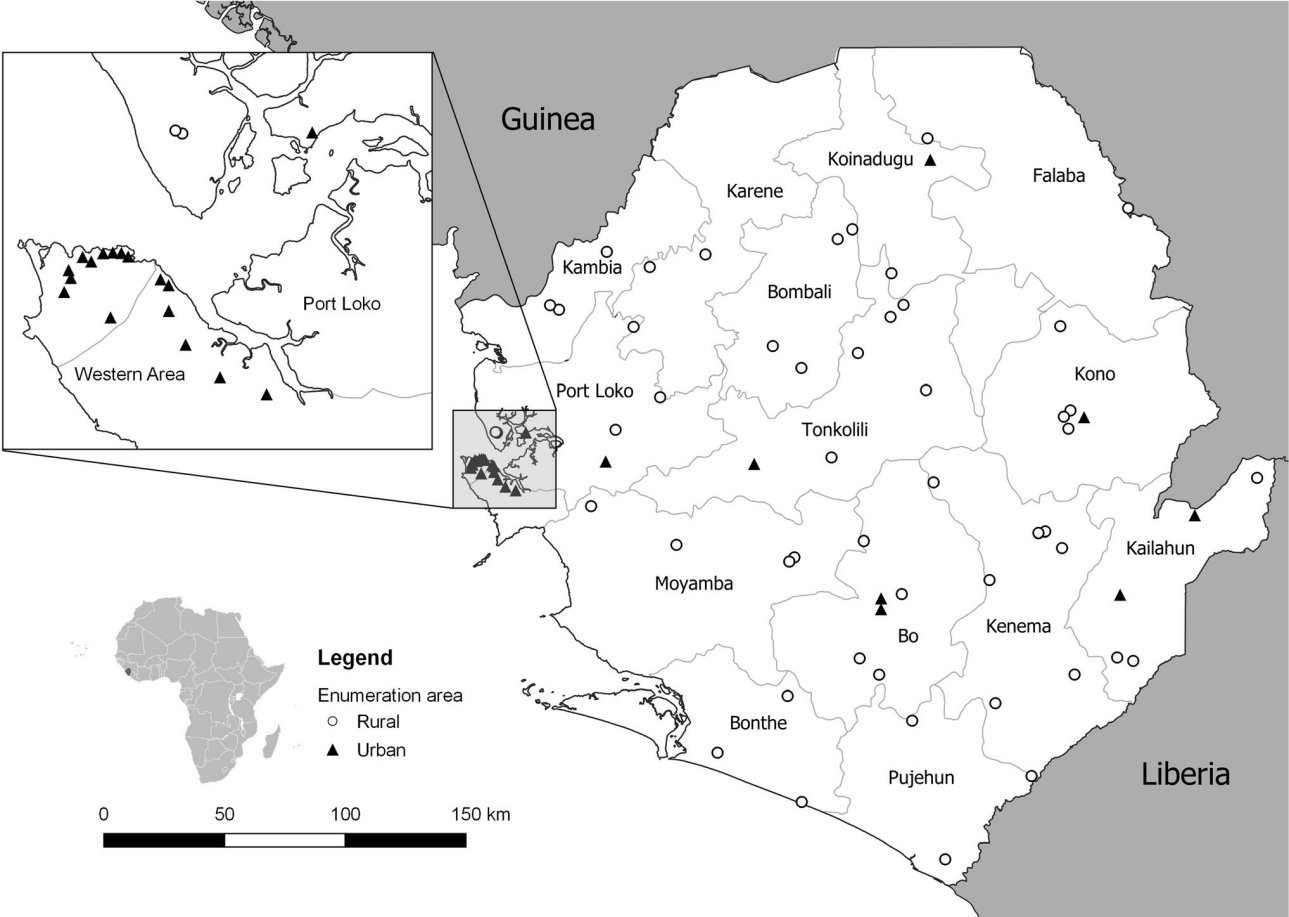
Distribution of the 75 rural and urban enumeration areas distributed across all 16 districts of Sierra Leone

**Fig. 2:**
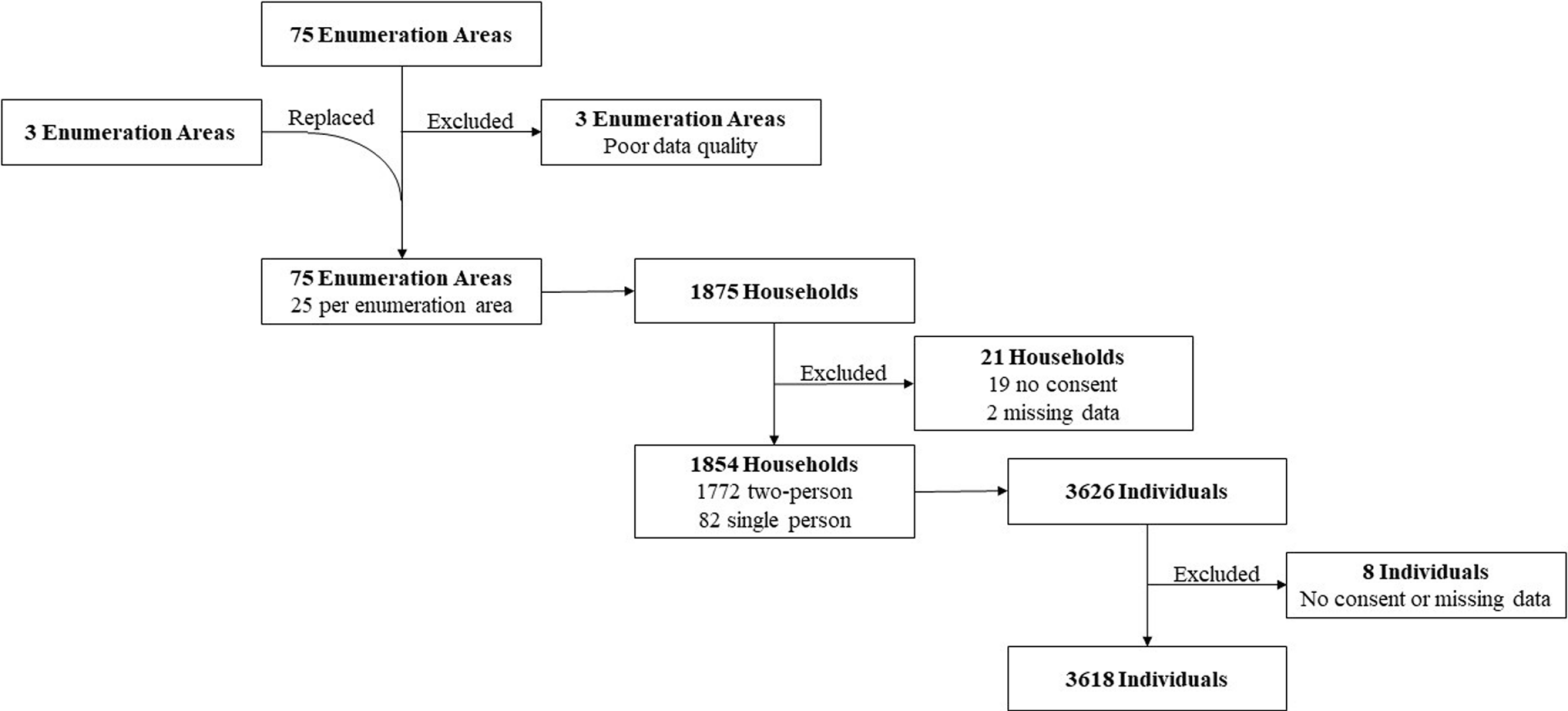
PRESSCO 2020 flowchart providing an overview in the course of 75 enumeration areas with 25 households each to ultimately 3618 individuals that were included for analysis

In total, 75 EAs (Fig. 1) were visited between October and November 2019 and between February and March 2020; the 3-month pause was because of a Lassa fever outbreak. Three EAs were substituted as data entries were missing upon checking for completeness by the data quality team. In total, 3618 household members from 1854 households were included for analysis (Fig. 2).

### Comparison with SOSAS (2012)

Compared to SOSAS, PRESSCO 2020 included a larger proportion of rural (66.9 vs 61.2%, *p* < 0.001) households with a smaller household size (mean 5.2 vs 6.4 individuals, *p* < 0.001) [2]. The distribution of other baseline data did not significantly differ (Table 1). In total, 278 (7.7%, 95% CI 6.8–8.6%) individuals reported 308 surgical conditions ranging from at least having acquired one wound, burn, mass, deformity, to having undergone at least one surgical procedure within the previous year. In Sierra Leone, this means an estimated 564,000 to 713,800 individuals have a surgical condition. At the time of the interview, 223 (6.2%, 95% CI 5.4–7.0%) individuals reported 255 conditions in need of surgical care. Compared to Groen et al. [2] the present study observed the prevalence of individuals reporting one or more possible surgical conditions decreasing from a reported 25% to 6.2%.

**Table 1:** Comparison of household characteristics and demographic data of PRESSCO 2020 and SOSAS conducted in 2012. PRESSCO 2020 is Prevalence Study on Surgical Conditions 2020. SOSAS is Surgeons OverSeas Assessment of Surgical needs

	PRESSCO 2020	SOSAS 2012	PRESSCO 2020 *vs.* SOSAS 2012
Household data			
Respondents	1854/1875 (99%)	1843/1875 (98%)	
Geographic Area			* p*< 0.001*
Urban	613/1854 (33%)	715/1843 (39%)	
Rural	1241/1854 (67%)	1128/1843 (61%)	
Household Head			* p* = 0.148
Male	1219/1854 (66%)	1253/1843 (68%)	
Female	635/1854 (34%)	590/1843 (32%)	
Mean household size (SD)	5.2 (2.6)	6.4 (5.3)	* p* < 0.001 *
Individual data			
Respondents	3618/3708 (98%)	3645/3686 (99%)	
Sex			* p* = 0.284
Males	1709/3618 (47%)	1677/3645 (46%)	
Females	1909/3618 (53%)	1968/3645 (54%)	
Median age (range)	20 (0 to 100)	20 (0 to 100)	* p* = 0.156
Ethnic origin			* p* = 0.172
Mende	1233/3618 (34%)	1309/3645 (36%)	
Temne	1003/3618 (28%)	1035/3645 (28%)	
Other	1382/3618 (38%)	1301/3645 (36%)	

Of the 255 physical conditions distributed over six anatomical locations, the most commonly reported surgical conditions were hard and soft masses (85; 33.3%), wounds (72; 28.2%), and acquired deformities (63; 24.7%) (Table 2). Compared to 2012, we documented that both fewer surgical conditions were reported, and a difference in distribution and anatomical location of the reported conditions *(p* < 0.001). Of the 308 conditions that existed during the last 12 months, 38 (12.3%) left the respondents incapacitated, caused shame (16; 5.2%), or required assistance with daily activities (5; 1.6%) or during transport (2; 0.6%) (Supplementary Table).

**Table 2:** Reported conditions and anatomical location of conditions needing surgical care. PRESSCO 2020 reported on 255 surgical conditions (vs. 1855 surgical conditions for SOSAS 2012) at the time of the interview

Reported condition	PRESSCO 2020	SOSAS 2012	PRESSCO 2020 *vs.* SOSAS 2012
Acquired deformity	63 (25%)	443 (28%)	< 0.001
Mass (hard and soft)	85 (33%)	423 (27%)	
Abdominal distention or pain	7 (3%)	190 (12%)	
Wound (injury related)	40 (16%)	179 (11%)	
Wound (not injury related)	32 (13%)	125 (8%)	
Burn	3 (1%)	86 (5%)	
Congenital deformity	15 (6%)	47 (3%)	
Urological complaints	5 (2%)	32 (2%)	
Bleeding from rectum	0 (0%)	24 (2%)	
Obstructed delivery	3 (1%)	22 (1%)	
Recurrent discharge from arms, hands, legs, or feet	2 (< 1%)	7 (< 1%)	
Missing	0 (0%)	7 (< 1%)	
Reported anatomical location	PRESSCO 2020	SOSAS 2012	PRESSCO 2020 *vs.* SOSAS 2012
Abdomen	20 (8%)	392 (25%)	< 0.001
Head, face, neck	63 (25%)	337 (21%)	
Arms, hands, legs and feet	99 (39%)	335 (21%)	
Groin, genitals, buttocks	51 (20%)	227 (14%)	
Chest, breast	4 (2%)	157 (10%)	
Back	18 (7%)	137 (9%)	
Total	255	1585	

### Surgical conditions

Surgical conditions were more common among men (9.7 vs 5.9% among women, *p* < 0.001) and predominantly reported among people older than 18 years. Need for a surgical consultation was equally distributed among members of rural and urban communities (7.8 vs 7.5%; *p* = 0.842) (Table 3).

**Table 3:** Health-seeking behavior in relation to sex, age and village type of the respondent

	All participants	Surgical condition*	Health seeking behavior**		Received surgical care***		
			Traditional healer	Health facility	Minor surgery	Major surgery	No surgical care
	*n*	*n* (%)	*n* (%)	*n* (%)	*n* (%)	n (%)	n (%)
Overall	3618	278 (7.7%)	53 (19.1)	210 (75.5)	149 (71.0)	33 (15.7)	25 (11.9)
Sex		*p* < 0.001	*p* = 0.213	*p* = 0.888	*p* = 0.352	*p* = 0.249	*p* = 0.230
Male	1709	166 (9.7)	36 (21.7)	126 (75.9)	86 (68.3)	23 (18.3)	17 (13.5)
Female	1909	112 (5.9)	17 (15.2)	84 (75.0)	63 (75.0)	10 (11.9)	8 (9.5)
Age		*p* < 0.001	*p* = 0.250	*p* = 0.022	*p* = 0.070	*p* = 0.128	*p* = 0.109
0–5 years	642	21 (3.3)	1 (4.8)	18 (85.7)	14 (77.8)	1 (5.6)	3 (16.7)
6–17 years	967	62 (6.4)	14 (22.6)	37 (59.7)	29 (78.4)	5 (13.5)	1 (2.7)
18–49 years	1481	141 (9.5)	25 (17.7)	112 (79.4)	83 (74.1)	15 (13.4)	14 (12.5)
50 years and older	508	51 (10.0)	12 (23.5)	41 (80.4)	22 (53.7)	12 (29.3)	6 (14.6)
Missing	20	3 (15.0)	1 (33.3)	2 (66.7)	1 (50)	0 (0.0)	1 (50.0)
Rural and urban		*p* = 0.842	*p* = 0.003	*p* = 0.765	*p* = 0.746	*p* = 0.221	*p* = 0.820
Rural	2432	189 (7.8)	45 (23.8)	144 (76.2)	101 (70.1)	26 (18.1)	18 (12.6)
Urban	1186	89 (7.5)	8 (9.0)	66 (74.2)	48 (72.7)	7 (10.6)	7 (10.6)

From 3618 household members complete datasets were available

*Individuals with a surgical condition in the last 12 months

**All with surgical condition in the last 12 months is used as denominator

***All participants that went to a health facility is used as denominator

### Seeking and receiving surgical care

Of the 278 individuals with at least one possible surgical condition, the majority visited a health facility (210, 75.5%). People living in rural areas were more likely to visit a traditional healer compared to those from urban areas (23.8 *vs*. 9.0%, *p* = 0.003). Of the 210 respondents visiting a health facility for their suspected surgical condition, 33 (15.7%) and 149 (71.0%) underwent major and minor surgery, respectively.

### Reasons for not seeking or receiving surgical care

For 80 (26%) of the 308 reported conditions that occurred during the past year, no care was sought or could be obtained despite visiting a health facility. Insufficient financial resources (48, 60.0%) were the main reason why no care was sought or obtained. Other reasons were a perception that the condition did not require surgical care (19, 23.8%) or lack of confidence in the health system (4, 5.0%) (Supplementary Table).

### Deaths

A total of 354 (179 men and 174 women) deaths during the 12 months preceding the survey were reported from 294 households. Based on a mean household size of 5.2, the crude death rate was 36.6 per 1000 population/year (Table 4).

**Table 4:** Household specific data on deceased household members. A total of 354 (179 men and 174 women) deaths during the year preceding the survey were reported from 294 households

	Deceased household members
Visited health facility in month before death	
Yes	284/353 (80%)
No	65/353 (18%)
Unknown	4/353 (1%)
Received surgical care	
No surgical care	310/353 (88%)
Major procedure	11/353 (3%)
Minor procedure	15/353 (4%)
Unknown	17/353 (5%)
Death in relation to last (major) surgical procedure	
< 1 week	6/11 (55%)
< 1 month	1/11 (9%)
> 1 month	4/11 (36%)

## Discussion

Following SOSAS that was performed in Sierra Leone and subsequently has been administered in other LMICs [2, 19–22], PRESSCO 2020 is the first surgical household survey that can compare its data to an earlier version of the same study. Nearly a decade after its initial surveillance, and 6 years after the onset of the West African EVD outbreak, the number of individuals reporting one or more possible surgical conditions was 6.2%, compared with 25% in 2012 [2]. A prevalence consistent with similar studies from Uganda (10.6%) and Nepal (10.0%), of which the latter survey included a visual physical examination [21, 22]. A validation study from northern Rwanda on a community-based survey including physical examination found an overall specificity of 97.7% for the structured interview [23]. The implied conformity between respondents without reported surgical conditions and absent findings by physical examination suggests that an underestimation of the reported prevalence is less likely.

To track progress on the development of the rapidly changing surgical ecosystem, PRESSCO 2020 re-applied a previously validated questionnaire [22]. When compared to one-time measurements, repeated household surveys yield finer granular data on the characteristics and situations of populations in need of surgical treatment [24]. Repeated household surveys generate timely data for program outcome monitoring and supplement hospital data to gain a better understanding of progress toward universal health coverage and the surgical volume indicator of the Lancet Commission on Global Surgery [1, 25, 26]. PRESSCO 2020 was developed in collaboration with members of SOSAS and carried out in collaboration with Stats SL. Our local adaptations on the SOSAS tool did not result in an increment of study costs [2]. The strengths of this study are that interviews and examinations were conducted by trained and local healthcare personnel and data checking and validation was carried out by a data quality team. The expansion of Sierra Leone’s telephone network during the interim period also facilitated the ease of transmission and verification of study data [27]. However, flaws during data collecting or misreporting of conditions that led to an overestimating of conditions in 2012, or underestimation of the true burden of surgical conditions in 2020 cannot be excluded.

### Interpretation

Compared to 2012, attention on access to essential surgical services has increased [25, 26]. However, an unequivocal explanation for the observed decrease in the surgery-requiring physical conditions is hard to provide. PRESSCO’s 2020 additional survey topics, physical examination, expanding from one to three enumerator teams, a total data gathering increase from three to seven weeks, but also more rigorous data checking procedures were the main methodological differences between the studies. Furthermore, except for Malawi, the prevalence of surgical conditions among other SOSAS-derived studies was more in line with the present findings [19–22]. Across other SOSAS publications, there are, however, discrepancies in the way results are presented, which renders direct comparisons difficult [19–22]. Four years after SOSAS Rwanda, a comparable survey also including a physical examination was repeated in northern Rwanda. A 12% prevalence of surgically treatable conditions was found among all examined individuals [28]. While more consistent with our finding, Rwanda has a higher HDI ranking (160 *vs.* 182 for Sierra Leone), and 70–80% of the population are enrolled in a community-based health insurance, covering in-and-outpatient services [14, 29]. This could suggest that our reported prevalence might lean on the low side; however, the data concerned came from only one district in Rwanda, and comparability of each dataset is unclear.

The gradual annual increase in health spending per capita might have been another contributing factor to the reduced surgical need. That notwithstanding, expenditures during and after the EVD outbreak were mainly due to external aid [30], and out-of-pocket payments remained responsible for roughly 45% of all healthcare-related expenses [31]. This is despite the rollout of the Free Health Care Initiative (FHCI) for pregnant women, lactating mothers, under five children and recently EVD survivors [11, 32]. If FHCI did not exist, 66.8% of women in Sierra Leone would experience catastrophic health expenditures [33]. As 60% of the reasons provided for not seeking health care were financial concerns, expanding the FHCI beneficiaries’ group [34], providing in-hospital meals for patients, facilitating road access to health clinics, and initiating a community-based health insurance are important [35].

Health system-strengthening initiatives [9–12], including the establishment of a task-sharing surgical training program in 2011 [8], nearly doubled the surgical workforce and enhanced the annual surgical volume by 15.6% to approximately 28.000 operations in 2017 [36]. Based on our findings, 86.7% of the people who visited the hospital actually underwent an operation. This high percentage suggests that people only visit the hospital when it is absolutely necessary. Consequently, 20% of the reported surgical conditions rendered respondents unemployed, disabled, or stigmatized—meaning that failure to amend surgical conditions in Sierra Leone has a negative effect on the country’s capacity to meet the SDG objectives; specifically, for inclusion, when so meeting the health needs of so many marginalized groups are a critical element to ending poverty [1, 37]. It is desirable to standardize collecting and reporting nationally representative data on surgical access [38]. Ideally, one validated surgical survey would be added as a special focus topic within the Demographic and Health Surveys (DHS) Program. In 2018, Zambia demonstrated that this was feasible [39].

## Limitations

A sub-analysis of the 2015 Global Burden of Disease study demonstrated a female/male ratio in the prevalence of surgical conditions of approximately 3:1 [40]. We found that surgical conditions were mostly reported by men. As men fall outside the FHCI beneficiaries’ group, this may contribute to their predominant presence among the respondents with a physical complaint. It is also plausible that healthy men could not be included as they worked away from home, which could have led to a selection bias. In comparison with SOSAS, the ranking of conditions and reported anatomical locations differed. These differences may be explained by minor differences in how patient’s history was taken, which could limit the internal validity of the survey. Lastly, the PRESSCO 2020 tool is not validated and surveys may not be as adept in explaining why people think or act as they do [41]. While surveys can tell how many respondents had a possible surgical condition or had surgery, they can be limited in the information they can provide in terms of personal reasons and sociocultural factors. It might have been better to answer these questions through qualitative research that is ethnographically informed [41].

PRESSCO 2020 is the first household survey into surgical need, which can compare its data to an earlier study. The reported prevalence was consistent with data from recent similar studies in other LMICs. Compared with 2012, the proportion of people with a surgical condition was found to be lower. Repeated household surveys related to health may help to properly measure and monitor surgery-related indicators in LMICs.

## Supplementary Information

Below is the link to the electronic supplementary material.

Supplementary file1 (DOCX 1863 kb)

Supplementary file2 (DOCX 17 kb)
