# Supplementary material for: PREvalence Study on Surgical COnditions (PRESSCO) 2020: A Population-Based Cross-Sectional Countrywide Survey on Surgical Conditions in Post-Ebola Outbreak Sierra Leone
Source: World J Surg. 2022 Sep 6;46(11):2585–94. doi: 10.1007/s00268-022-06695-7 (PMC9529684; doi:10.1007/s00268-022-06695-7)
Supplement: Supplementary file 2 — Supplementary file2 (DOCX 17 kb) [file 268_2022_6695_MOESM2_ESM.docx]

**Supplementary Table.** Impact of surgical condition on daily life and reasons for not seeking or receiving surgical care. Top table provides an overview on the impact on daily life for each of the 308 reported conditions during the past year. For 80 out of 308 conditions, no care was sought or received; bottom table.

| **Supplementary table** | |
| --- | --- |
| **Impact on daily life per condition** | n (%) |
| Condition is not disabling | 247 (80) |
| I'm not able to work anymore | 38 (12) |
| Condition causes shame | 16 (5) |
| I need assistance with day to day activities | 5 (2) |
| I need assistance with transport | 2 (1) |
| Total | 308 |
| **Reasons** | n (%) |
| Unable to pay for care (incl. transport) | 48 (60) |
| No perceived need | 19 (24) |
| Not specified | 6 (8) |
| Shame, fear or lack of trust | 4 (5) |
| Services not available | 2 (3) |
| Under care of traditional healer | 1 (1) |
| Total | 80 |
